# Miniaturized Wideband Antenna Prototype Operating over the Ku-Band

**DOI:** 10.3390/mi13030471

**Published:** 2022-03-19

**Authors:** Sujan Shrestha, Hijab Zahra, Arslan Kiyani, Mohsen Asadnia, Syed Muzahir Abbas, Abdelhady Mahmoud

**Affiliations:** 1 School of Engineering, Faculty of Science and Engineering, Macquarie University, Sydney, NSW 2109, Australia; hijab.zahra@hdr.mq.edu.au (H.Z.); arslan.kiyani@mq.edu.au (A.K.); mohsen.asadnia@mq.edu.au (M.A.); syed.abbas@mq.edu.au (S.M.A.); 2 BENELEC Technologies, Botany, NSW 2019, Australia; 3 Faculty of Engineering, Benha University, Benha 13512, Egypt; abdoeng78@gmail.com

**Keywords:** wideband operation, microwave communication, 3D printing

## Abstract

A wideband antenna is proposed based on three-dimensional printing technology. The antenna was designed using the PREPERM 10 material, with permittivity $\epsilon_{r}$ = 10, where the overall height of the proposed prototype was maintained as 12.83 mm (0.51λ), having a lateral dimension of 60 mm × 60 mm, at an operating frequency of 12 GHz (λ = 25 mm). The proposed antenna achieved a wide frequency bandwidth with a voltage standing-wave ratio (VSWR) of less than two, from 10 GHz to 15 GHz in the Ku-band, where the maximum directivity was 20 dBi over a reflection coefficient bandwidth of 50%. It showed a miniaturized non-uniform metasurface of 2.4λ × 2.4λ × 0.51λ that was placed at 16.5 mm (0.66λ) above the ground plane, which was 2.4λ × 2.4λ × 0.04λ in dimension. Thus, the overall height of the proposed antenna system from the feed source was 29.33 mm (1.17λ). The total weight of the system including the designed structures made of PREPERM 10 and ABS with copper-painted prototypes was 96 g and 79 g, respectively. The measured results were consistent with the simulated results, demonstrating the feasibility and effectiveness of the proposed method.

## 1. Introduction

The need for high-data-rate, compact-size, and low-power-consumption antennas is a growing area of study, in particular for wideband communication systems, which depend on the efficient design of such antennas [1]. In order to maintain efficient voice and video transmission, there is a requirement for a large bandwidth with a higher gain. Dielectric lens [2] antennas show those characteristics with simple feeding methods. However, those lenses are higher in profile and a have bulky size. Similarly, reflect array antennas [3,4,5] and transmit array antennas [6,7] generate wideband characteristics that depend in particular on the ratio of the focal distance to the diameter of the antenna’s aperture, as well as on the designed patches on the substrate layers. This will ultimately increase the overall antenna height, and those structures show greater aperture dimensions. In order to address such issues for wide bandwidth operation, several designed superstrates have been studied. The use of substrate layers was shown in [8,9,10] in the design of a single-layer substrate with diverse materials [11], and partially reflecting surfaces (PRSs) designed with a single dielectric material [12] were studied. Those prototypes utilize expensive dielectric slabs using a standard ceramic cutting process, which are bulky in nature. Most recently, metasurfaces were studied that were electrically thin and showed a periodic structure, which were implemented in a printed circuit board (PCB). A compact low-cost PRS having a non-uniform double-sided printed single-layer dielectric slab was proposed in [13]. Similarly, different shapes of unit cell patch designs were studied in [14,15,16,17,18,19], where the use of electrically thin substrates with the respective designed patches was shown. This further shows the design complexity with a complex geometry, which results in an increased fabrication cost. Thus, in addressing these issues, the impact of 3D printing is today being felt far beyond the metal industry. This is unlocking new applications where there is a need for a complex geometrical structure, which was previously impossible. A plastic body of the antenna was designed through 3D printing techniques and later coated with commercially available spray coating [20,21,22], which showed the improvement of the antenna gain. Similarly, a 3D-printed structure that utilized diverse infill ratios was proposed in [23], which was able to show improved directive radiation characteristics. Additionally, the broadband response was achieved through the use of alternative dielectric layers with both low- and high-permittivity materials, which were implemented through plastic filaments realized by Fused Deposition Modeling (FDM) and with ceramic material such as alumina, respectively [24]. The 3D-printed antenna showed effective performance in increasing the performance of the feed source [25,26], as well as in the beam tilting phenomenon [27]. In light of employing a low-profile and low-cost 3D-printed antenna design that utilizes a single material, this article proposes a miniaturized metasurface antenna prototype that consists of non-uniform cuboids, which have a rectangular base as a support structure. The novelty of the proposed system lies in the elimination of expensive substrates, demonstrating a cost-effective solution for wideband antenna design. The Ku-band-operated antenna prototype is applicable in the fields of microwave and satellite communication links [28,29,30]. The proposed antenna design was verified by simulation using CST Microwave Studio [31] and compared against the experimental data. The article is organized as follows: Section 2 deals with the design aspect of the cylindrical unit cell, which highlights the magnitude and phase plots along with the proposed 3D printable structure. Alongside this, it also depicts the phase distribution noted above of the designed structure. Section 3 details the fabrication technology and the radiation plots. Finally, concluding remarks are given in Section 4.

## 2. Design of the Unit Cell

**Figure 1:**
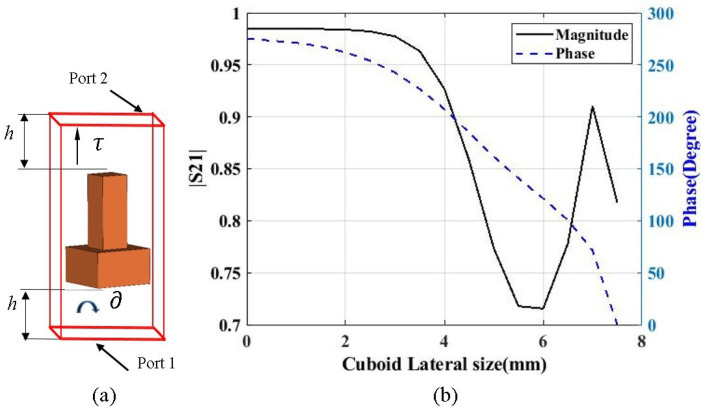
(**a**) Unit cell with two-port networks. (**b**) Generated transmission magnitude and phase plots.

This section details the design concept of the cylindrical unit cell in a two-port network. Figure 1a shows the analyzed unit cell. The cuboids were 8.33 mm (0.3λ) in height and were held by a 7.5 mm × 7.5 mm × 4 mm (0.3λ × 0.3λ × 0.16λ) rectangular base whose overall dimensions were 7.5 mm × 7.5 mm × 12.33 mm (0.3λ × 0.3λ × 0.49λ). The lateral dimension of the cuboid changed from a minimum of 0.5 mm (0.02λ) to a maximum of 7.5 mm (0.3λ) at intervals of 0.5 mm, where the length and breadth had equal values. In two-port networks, port 1 and port 2 are located at the half wavelength, a h = 12.5 mm (0.5λ) distance from the base and top side of the unit cell. Port 1 was kept below the bottom surface at a λ/2 distance, and port 2 was kept above the top surface at a λ/2 distance, where the distance of the ports is indicated by h. In order to conceptualize the unit cell, we considered only the electric field component from the Field Monitors part of the Navigation Tree from CST MWS. The side faces of the unit cell comprised the electric field component.

Similarly, Figure 1b depicts the generated magnitude and phase plot of the unit cell. The transmission phase plots lie within 0.7 and 0.99, making the structure more transmissible with a smaller cuboid lateral dimension, in particular for values less than 4 mm. Additionally, the phase values also lied between $200^{\circ }$ and $280^{\circ }$. Interestingly, as the cuboids’ sizes increased, the corresponding phase decreased, but in contrast, the transmission magnitude increased from 0.72 to 0.92 and again decreased to about 0.82. This fluctuation was due to the higher permittivity values, which made the structure more reflective for higher cuboid sizes. We considered 12 GHz as the simulation frequency to design the proposed unit cell.

### 2.1. Generation of the Proposed Prototype

**Figure 2:**
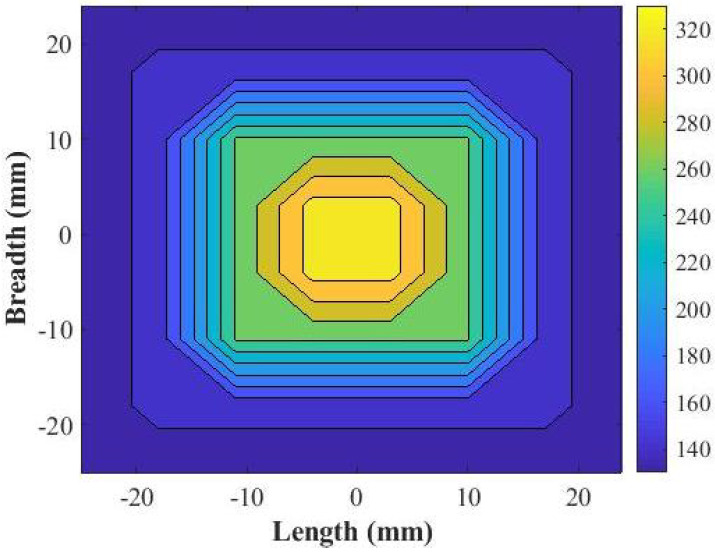
Distribution of the phase as noticed above the ground plane in the 12 GHz designed frequency.

In order to generate the proposed prototype, the phase distribution was noted near the center of the ground plane along the E-plane axis, which was connected to the feed waveguide, WR-75, which was less than one-eighth of the wavelength, 3 mm (0.12λ). The slot x-axis and y-axis at the center of the ground plane were maintained as 19.1 mm × 9.5 mm (0.76λ × 0.38λ), which were the same dimensions as the WR-75 slot. Figure 2 shows the noted phase distribution above the ground plane. The ground plane, which was 2.4λ × 2.4λ × 0.04λ in dimension, was square in shape. The phase values were noted across its aperture, starting from the center position, where it had relatively higher phase values, and across the end, these values decreased. We used the unwrapped E-field phase pattern obtained from CST MWS firstly at the center position, then successively, these E-field phase patterns were obtained for 3.75 mm, 11.25 mm, 18.75 mm, and 26.25 mm from the center of the aperture. These values were symmetrically arranged across the surface. A phase difference of $214.09^{\circ }$ was noticed as calculated with the difference of the phase from the center towards the aperture. At 3.75 mm, 11.25 mm, 18.75 mm, and 26.25 mm from the center of the aperture, the noted phase values were respectively $329.20^{\circ }$, $261.86^{\circ }$, $147.40^{\circ }$, and $125.90^{\circ }$.

In order to generate the proposed prototype, the design procedures are listed below:

Step 1: Start;
Step 2: Note the phase values above the feed waveguide source at 3 mm above it;
Step 3: Place the respective cuboids noted from the database of the transmission coefficient magnitude and phase values across the aperture positions;
Step 4: Check the corrected phase near the top surface of the proposed prototype at 0.125λ;
Step 5: Is the corrected phase value less than $120^{\circ }$ in the designed frequency?
If No, change the cuboids’ lateral dimensions, and again, place them in the defined aperture positions. Go to Step 4;
If Yes, place the respective cuboids in the defined aperture positions of 3.75 mm, 11.25 mm, 18.75 mm, and 26.25 mm from the center position. This will generate the proposed prototype;
Step 6: End.

**Figure 3:**
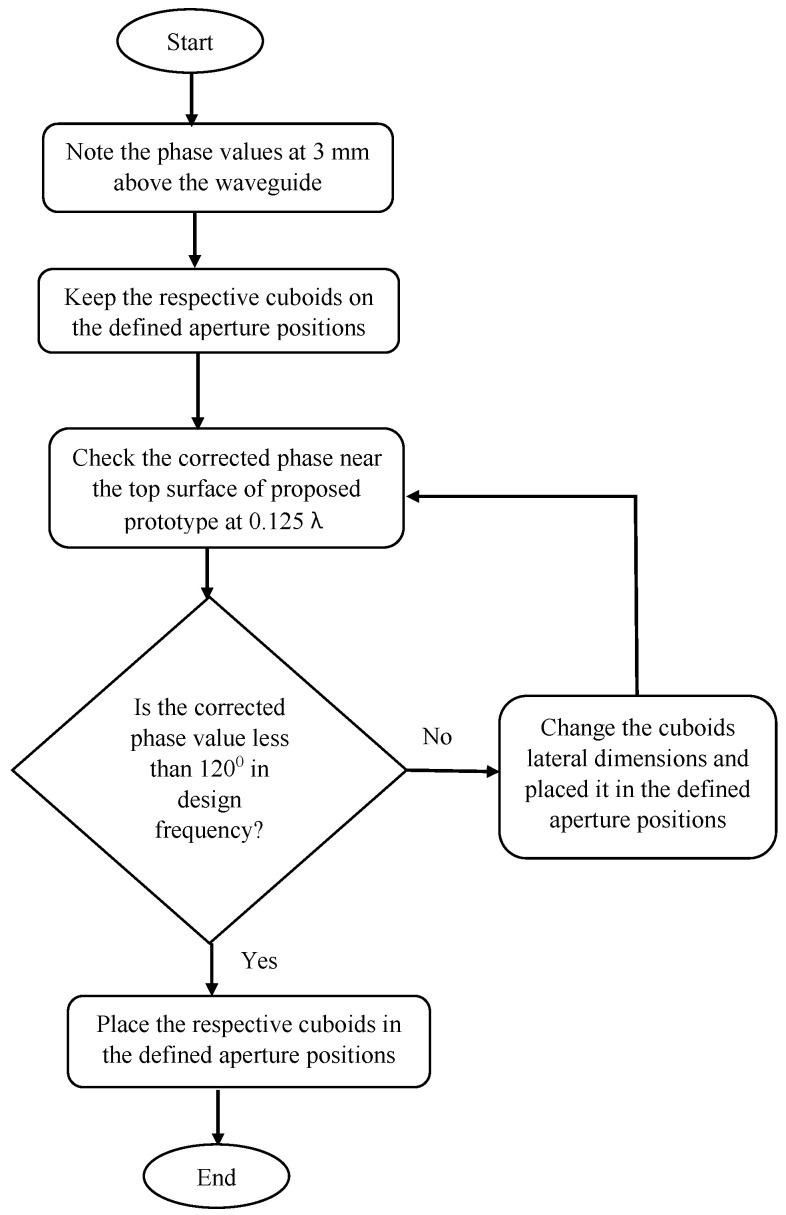
Flow diagram showing the generation of the proposed prototype.

The flow diagram of the design procedure is shown in Figure 3.

**Figure 4:**
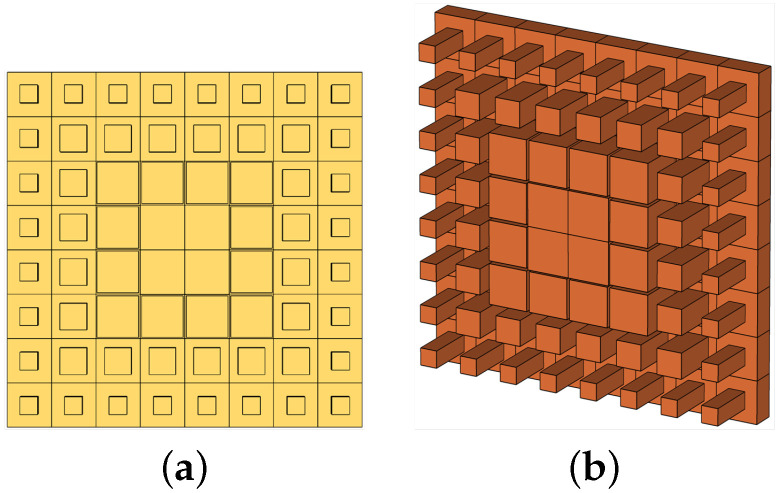
Pictorial views of the proposed prototype. (**a**) Top view. (**b**) Perspective view.

Thus, the noted phases were normalized considering the phase value as greater than the maximum, which would ultimately determine the respective cuboids that shall be placed at the respective distances of 3.75 mm, 11.25 mm, 18.75 mm, and 26.25 mm from the center of the aperture. From the data, as shown in Figure 1b, the respective phase values determined the corresponding cuboids’ lateral dimensions, which were 7.5 mm, 7 mm, 4.5 mm, and 4 mm, and were placed at the respective positions of 3.75 mm, 11.25 mm, 18.75 mm, and 26.25 mm from the center of the aperture. Successively, we slightly tuned the cuboids’ lateral dimension around the aperture, which was at a distance of 26.25 mm from the center, where its value decreased to 3 mm, and the overall height of the cuboids was maintained at 8.83 mm, as well as the cavity height at 16.5 mm. This was performed to obtain a higher directivity and gain of the antenna system, which shall ultimately generate a more uniform phase above the proposed metasurface. Hence, we had four different rounds of cuboid placement. Round 1, Round 2, Round 3, and Round 4 were considered respectively for 3.75 mm, 11.25 mm, 18.75 mm, and 26.25 mm from the center of the aperture. The obtained proposed metasurface is shown in Figure 4a,b, respectively the top and perspective views.

### 2.2. Phase Distribution of the Proposed Prototype

**Figure 5:**
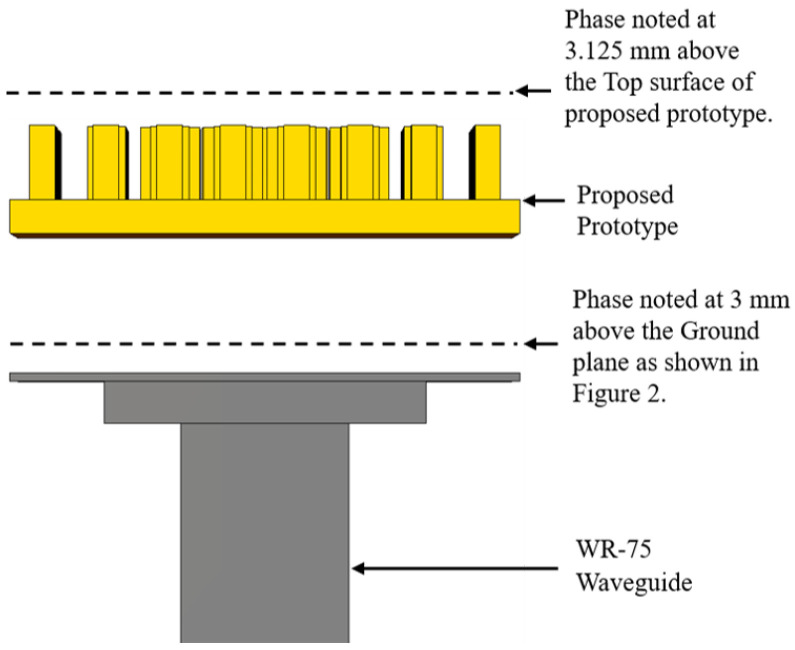
Overall system setup for the phase distribution.

**Figure 6:**
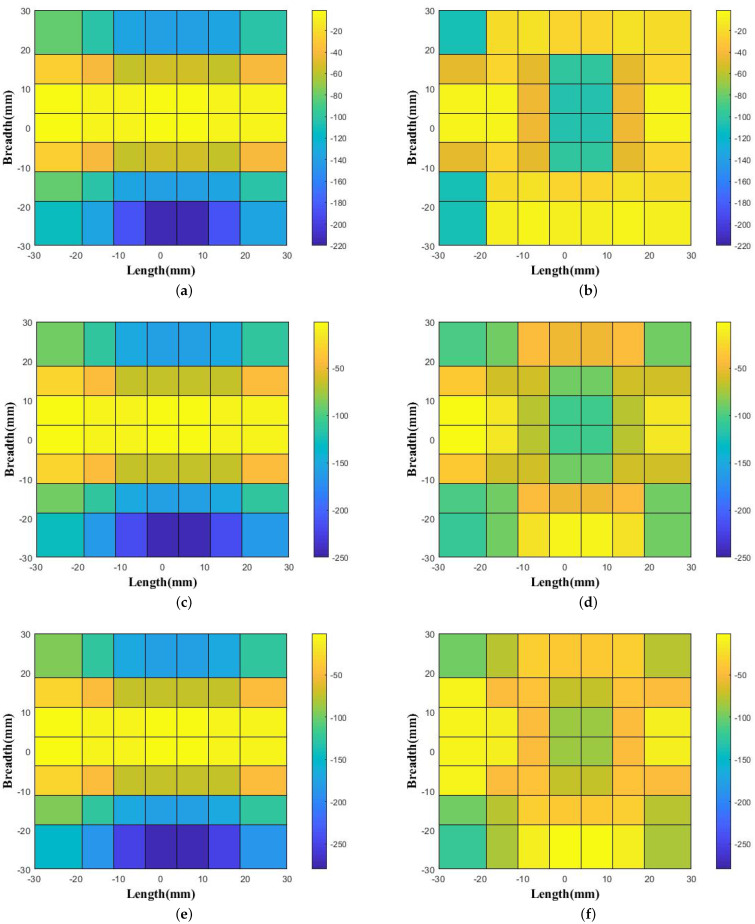
Obtained phase distribution noted above the feed WR-75 waveguide without the proposed prototype (**a**) at 10 GHz, (**c**) at 11 GHz, and (**e**) at 12 GHz and with the placement of the proposed prototype (**b**) at 10 GHz, (**d**) at 11 GHz, and (**f**) at 12 GHz.

**Figure 7:**
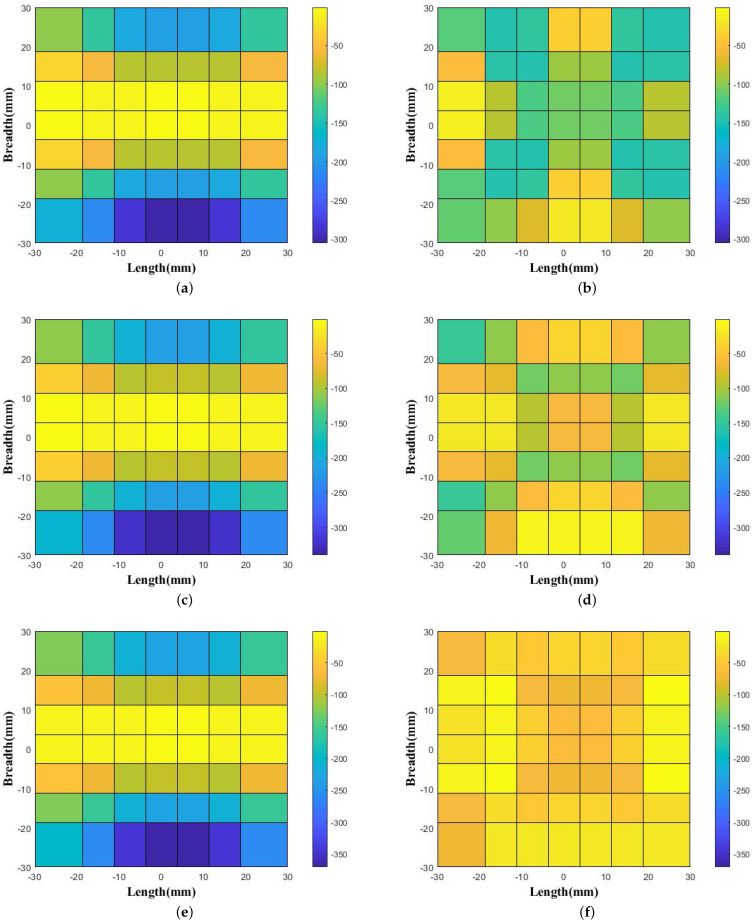
Obtained phase distribution noted above the feed WR-75 waveguide without the proposed prototype (**a**) at 13 GHz, (**c**) at 14 GHz, and (**e**) at 15 GHz and with the placement of the proposed prototype (**b**) at 13 GHz, (**d**) at 14 GHz, and (**f**) at 15 GHz.

The phase values were obtained from 10 GHz to 15 GHz at intervals of 1 GHz for two conditions. Firstly, the phase distributions were calculated just above the ground plane at a height of 3 mm, where there was no use of the proposed wideband prototype. The phase was noted very close to the source because of the relatively lower directivity and gain values of the feed waveguide. The phase variation could be better visualized if we note the phase distribution near the source. Secondly, the phase variation was calculated at 3.125 mm (0.125 λ) above the top surface of the proposed prototype as it was kept above the ground plane. The overall system configuration of the phase distribution is shown in Figure 5. Hence, the noted phase variations without and with the placement of the proposed prototype above the ground plane for 10 GHz, 11 GHz, and 12 GHz are shown in Figure 6a,c,e and Figure 6b,d,f, respectively. Similarly, the phase patterns for 13 GHz, 14 GHz, and 15 GHz without and with the placement of the proposed prototype above the ground plane are shown in Figure 7a,c,e and Figure 7b,d,f, respectively. The phase variations as calculated with the difference of the phase value from the center and the end aperture position for 10 GHz, 11 GHz, 12 GHz, 13 GHz, 14 GHz, and 15 GHz were respectively $211.74^{\circ }$, $240.65^{\circ }$, $270.45^{\circ }$, $299.97^{\circ }$, $329.53^{\circ }$, and $362.29^{\circ }$, which were calculated without the placement of the proposed wideband prototype. These values were further corrected, and more uniform phase variations were observed after the placement of the proposed prototype. Thus, the respective corrected phase values were $105.73^{\circ }$, $105.87^{\circ }$, $117.45^{\circ }$, $132.28^{\circ }$, $138.49^{\circ }$, and $65.70^{\circ }$ for frequencies of 10 GHz, 11 GHz, 12 GHz, 13 GHz, 14 GHz, and 15 GHz. The color bar was maintained for the same value range to show the correction thus obtained in the phase variation. Therefore, we depict the phase unit over the color bar with the same value range. The lattices in the two figures are not identically square because the phase values were corrected in a more uniform manner and the corrected phase values were less for the aperture positions. We show different phase distributions with respect to the frequency and material because the structure was wideband in nature, operating from 10 GHz to 15 GHz. In order to visualize the uniform phase distribution across the frequency band, we plot these phase values in Figure 6 and Figure 7.

## 3. Results and Discussion

This section details the fabrication and measurement setup. The obtained radiation patterns showed lower side lobe levels over the Ku-band frequency range, as well as the characteristics of the proposed antenna prototype, depicting its uniqueness as compared to other prototypes.

### 3.1. Details of the Fabrication and Measurement

**Figure 8:**
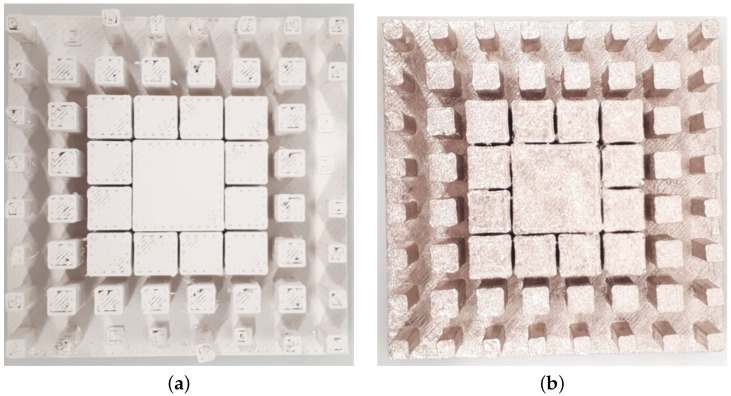
Fabricated prototype made (**a**) from PREPERM 10 and (**b**) from ABS with copper paint.

In order to achieve miniaturized Radio-Frequency (RF) parts, higher permittivity filaments are used, especially in the microwave region [32]. The filament used to fabricate the proposed prototype was the PREPERM 3D ABS1000 filament with a 1.75 mm thickness [33], which is based on Fused Deposition Modeling (FDM) and shows a relative permittivity $\epsilon_{r}$ = 10 and loss tangent value tanδ = 0.003. The Omni 3D printer was used for the fabrication process, where we maintained the infill ratio as 100% and manually changed the temperature and specific gravity value of the filament. Thus, the 3D printer was able to generate the required proposed antenna prototype. On the other hand, the study was performed by coating the 3D-printed parts in metal. We considered an Acrylonitrile Butadiene Styrene (ABS) filament for the Omni 3D printer with a 100% infill ratio. Next, the realized part’s surface was painted with copper. Hence, it was used above the ground plane and was fed by the WR-75 waveguide. The fabricated prototypes from PREPERM 10 and ABS painted with copper are shown in Figure 8a,b.

**Figure 9:**
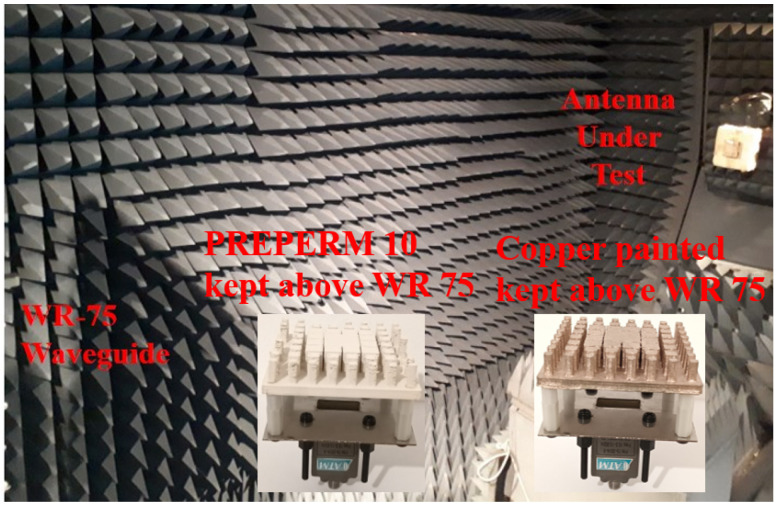
Experimental setup of the measurement system.

The measurement setup was performed in the NSI-700S-50 spherical near-field measurement system at the Australian Antenna Measurement Facility. The integration of the proposed prototypes along with the system setup is shown in the Figure 9, where the pictures show the prototypes kept above the WR-75 waveguide.

Hence, the measured results were compared against the simulated results as obtained from the commercial software CST Microwave studio. These are detailed in a further section that includes the radiation plots, directivity, gain, and Voltage Standing-Wave Ratio (VSWR). The proposed prototype was kept above the feed source, which was the WR-75. The overall system was able to show the improved properties of the antenna system after the placement of the proposed prototype just above the WR-75 where the ground plane acted as a reflective surface. The designed structure with PREPERM 10 and ABS with copper-painted surfaces showed the improved miniature wideband characteristics that were justified through the radiation patterns observed from 10 GHz to 15 GHz.

### 3.2. Radiation Plots

**Figure 10:**
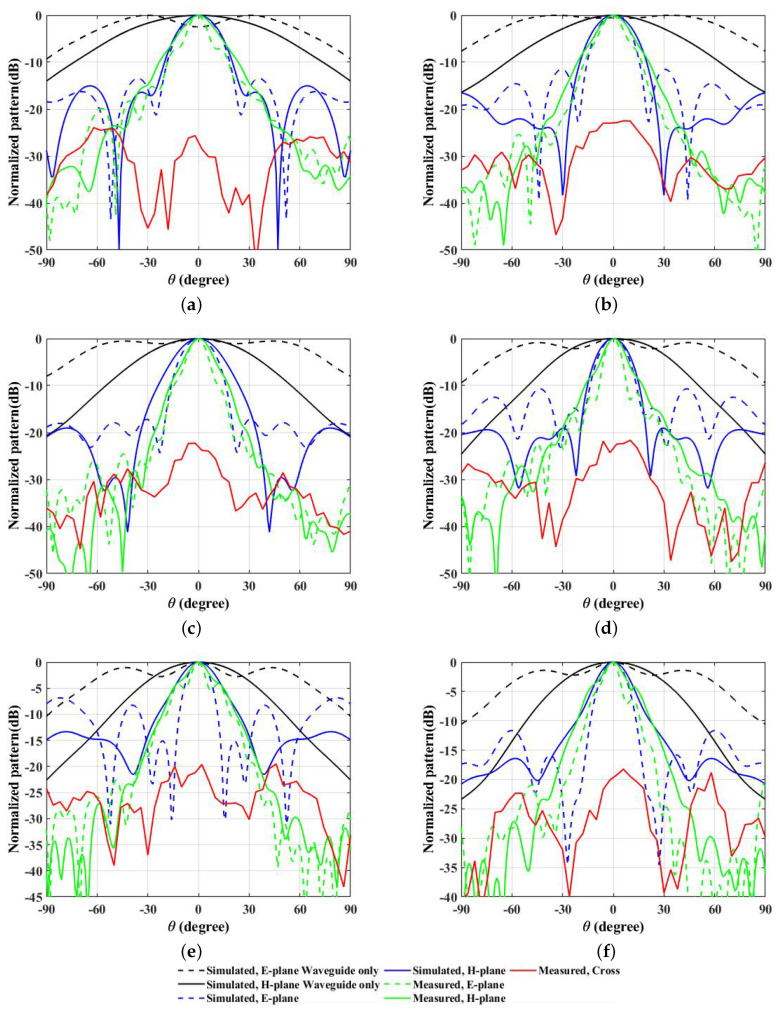
Simulated and measured radiation patterns as observed with the use of the PREPERM 10 designed surface, which is shown in (**a**–**f**) for 10 GHz, 11, GHz, 12 GHz, 13 GHz, 14 GHz, and 15 GHz frequencies.

**Figure 11:**
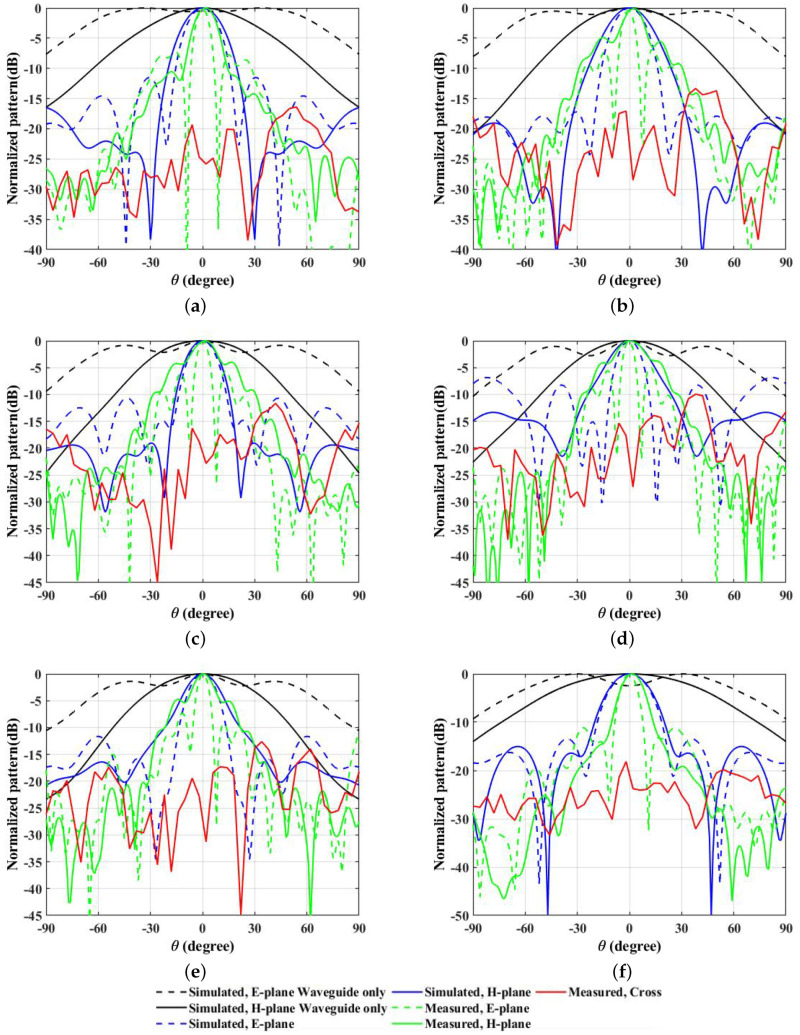
Simulated and measured radiation patterns as observed with the use of the copper-painted ABS designed surface, which is shown in (**a**–**f**) for 10 GHz, 11, GHz, 12 GHz, 13 GHz, 14 GHz, and 15 GHz frequencies.

The radiation patterns for the E-plane and H-plane with and without the proposed prototype as observed by the use of the PREPERM 10 surface design are shown in Figure 10a–f for 10 GHz, 11 GHz, 12 GHz, 13 GHz, 14 GHz, and 15 GHz frequencies, respectively. Similarly, the radiation patterns for the E- and H-plane with and without the proposed prototype obtained after the use of the copper-painted ABS designed surface are shown in Figure 11a–f for 10 GHz, 11, GHz, 12 GHz, 13 GHz, 14 GHz, and 15 GHz frequencies, respectively. The radiated beam patterns were more aligned with the use of PREPERM 10 designed surface as compared to the copper-painted ABS designed structure. This might be due to the resonating behaviors of the PREPERM 10 material, which has higher dielectric constant values. Interestingly, the beam width was relatively smaller when the copper-painted ABS structure was used above the WR-75 waveguide, which might be due to the structural design, where in the middle section, we could not paint the cylindrical units with copper as they were closer to each other. These figures show the directive radiation patterns with lower side lobe levels, where in the E-plane, they were −13.4 dB, −11.5 dB, −17.2 dB, −10.7 dB, −6.8 dB, and −11.6 dB and in the H-plane −15 dB, −16.2 dB, −19 dB, −19.1 dB, −13.3 dB, and −16.4 dB, respectively, for 10 GHz, 11, GHz, 12 GHz, 13 GHz, 14 GHz, and 15 GHz frequencies.

### 3.3. Plots of the Properties

**Figure 12:**
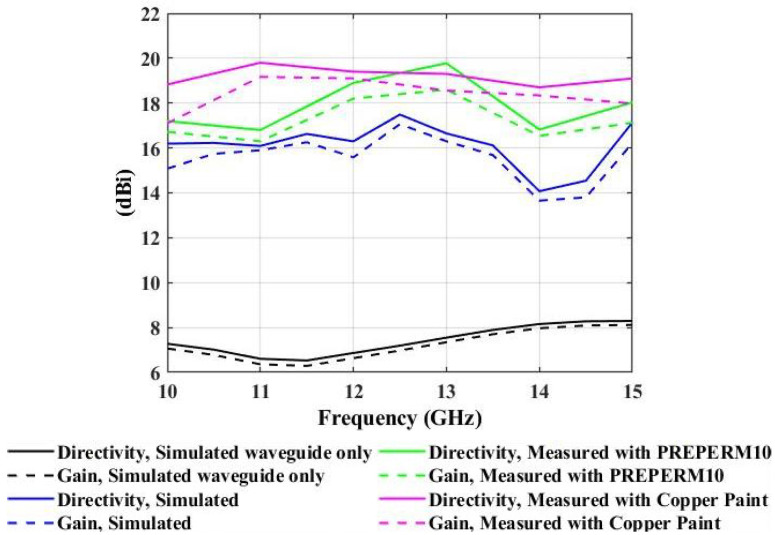
Simulated and measured directivity and gain as observed with the placement of the proposed miniaturized wideband prototype.

The proposed miniaturized wideband prototype showed higher directivity and gain, which were greater than 14 dBi and 13.7 dBi, respectively, in the operating frequency range from 10 GHz to 15 GHz. Interestingly, the measured directivity and gain values were above 16.8 dBi and 16.3 dBi for the PREPERM 10 designed surface and 18.7 dBi and 17.1 dBi for the ABS designed copper-painted structure, respectively. In both cases, the simulated results were compared against the measured values, as shown in Figure 12. This figure highlights the wideband nature of the proposed designed surfaces.

**Figure 13:**
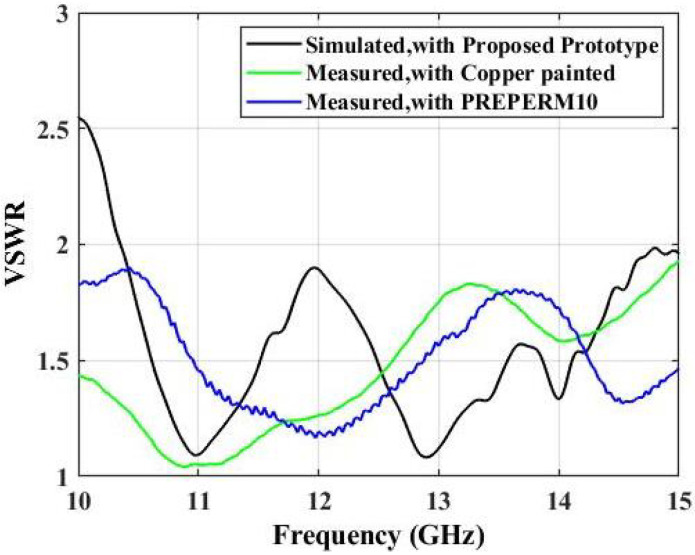
Simulated and measured VSWR plots.

The input simulated and measured impedance patterns are presented in Figure 13. The values were less than 2 over the operating frequency range. As noticed, the surfaces designed from PREPERM 10 and copper-painted ABS both had values less than 2, which signified the wideband nature of the proposed prototypes. We used ABS filament to realize the 3D-printed structure of the designed surface, which was later painted with copper. As the copper painting was performed on the surface of the 3D-printed structure, this prototype was still able to transmit EM waves, as shown by the experimental results in the radiation patterns. These patterns were more directive in nature and closely matched the radiation plots. The obtained matching values were below 1.8 throughout the operating frequency band, which signified that the structure was able to transmit EM waves.

Comparison between the proposed antenna and the already presented antennas based on the design concept and material substrate used is presented in Table 1. The comparison results showed that the proposed antennas had a wider bandwidth, better gain, and a miniature aperture and were lighter in weight compared to the reported antennas. Additionally, the use of expensive substrates could be minimized after the application of the proposed prototype.

## 4. Conclusions

This paper presented the development of a simple, miniaturized, wideband antenna prototype. The proposed antenna prototype was printed using a single material, the Premix PREPERM 3D ABS1000 filament for FDM 3D printing, where the variation in the lateral dimension of the cuboids resulted in different phase values, which ultimately showed wideband characteristics. Additionally, the same prototype was realized by using ABS filament, which was copper coated. The manufactured prototype had 64 cuboids with the variation of its lateral dimension from the center towards the aperture, where the central cuboids had relatively higher dimensions as compared to the cuboids at the end of the aperture. The 3D-printed PREPERM 10 structure and copper-painted ABS prototype showed a directivity of 16.8 to 19.78 dBi and 18.7 to 19.8 dBi and a gain of 16.3 to 18.6 dBi and 17.1 to 19.17 dBi, respectively, where the side lobe levels were less than −11.5 dB over the operating frequency range from 10 GHz to 15 GHz, when fed by the WR-75 waveguide, which was attached to the ground plane, which was located at 16.5 mm (0.66λ) below the lower surface of the proposed antenna prototypes. The ground plate had central slot dimensions of 19.1 mm × 9.5 mm (0.76λ × 0.38λ), which were equal to the internal slot dimensions of the WR-75 waveguide. Additionally, the obtained directivity and gain values were above 16.8 dBi and 16.3 dBi for the PREPERM 10 designed surface and 18.7 dBi and 17.1 dBi for the copper-painted ABS designed structure, respectively. We emphasize the wideband nature of the proposed designed structures. The total weights of the system including the designed structures made from PREPERM 10 and copper-painted ABS were 96 g and 79 g, respectively, which signified that they were light in weight compared to the other antenna structures.

## Figures and Tables

**Table 1:** Comparison of the proposed wideband prototypes against the available antenna structures.

Ref.	Operating Bandwidth (GHz)	Electrical Area (mm × mm)	Electrical Height from Feed (mm)	Lowest Operating Frequency (GHz)	Operating Frequency (GHz)	Peak Gain (dBi)	Peak Directivity (dBi)	Bandwidth (%)	3dB Bandwidth (%)	Side Lobe Level (H-Plane) (dB)	Side Lobe Level (E-Plane) (dB)	Polarization	DBP/A	Thickness of Substrate (mm)	Aperture Size	Fabrication Technique	Weight (Grams)
[8]	10 to 15	1.5λ (40.5) × 1.5λ (40.5)	0.5λ (13.5)	10	11.1	18.2	18.7	30	21.7	−19 in 12.6 GHz	−16.9 in 12.6 GHz	Linear and circular	n/a	0.52λ (14.05)	1.5λ × 1.5λ × 0.52λ	Roggers TMM3, RT/duroid 6010.5, Roggers TMM4	n/a
[11]	10 to 17.2	1.54λ${}^{2}$ (Radius = 27 mm)	0.5λ (13.5)	10	11.11	16.3	16.4	52.94	54.2	−12 to −8	−15 to −10	Linear	1501	0.28λ (7.62)	1.54λ${}^{2}$ × 0.28λ	Rogers RT 6010.2, RT6006, TMM3	n/a
[13]	10.1 to 14.9	3.8λ${}^{2}$ (Radius = 29.25 mm)	0.55λ (14.48)	10.1	11.4	15.75	16.2	38	21.5	n/a	n/a	Linear	n/a	0.12λ (1.52)	3.8λ${}^{2}$ × 0.12λ	Roggers TMM4 slab	n/a
[15]	Ku−band (11.7 to 19.8)	1.75λ (35) × 1.75λ (35)	0.475λ (9.5)	11.7	15	11.45	n/a	54	42.3	n/a	n/a	Circular	n/a	0.15λ (3)	1.75λ × 1.75λ × 0.15λ	Period metal patches Rogers DT/duroid 5880	n/a
[16]	X-band (8.88 to 11.25)	1.98λ (66) × 1.98λ (66)	0.45λ (15)	8.88	9	13.92	n/a	25.1	23.6	n/a	n/a	n/a	n/a	0.024λ (0.787)	1.98λ × 1.98λ × 0.024λ	Period metal patches Rogers RT/duroid 5880	n/a
[17]	8.6 to 11.4	2.4λ (72) × 2.4λ (72)	0.5λ (15)	8.6	10	13.8	16.5	28	28	n/a	n/a	n/a	n/a	0.026λ (0.787)	2.4λ × 2.4λ × 0.026λ	Period metal patches Rogers RT/duroid 5880	n/a
[18]	8.2 to 12.5	1.5λ (45) × 1.5λ (45)	0.6λ (20)	8.2	10	13	13.2	44	46	n/a	n/a	Linear	n/a	0.8λ (24)	1.5λ × 1.5λ × 0.8λ	FR4 substrate with metal patches	n/a
[20]	X−band (λ = 26.55 mm)	3.85λ (102) × 2.7λ (72)	n/a	10	11.3	18.7	n/a	38	9.7	−20 in 11.3 GHz	−13.5 in 11.3 GHz	n/a	n/a	0.45λ (12)	3.85λ × 2.7λ × 0.45λ	Vero clear material	195
[21]	Ku−band (10.5 to 14.5)	0.785λ${}^{2}$ (Radius = 70 mm)	0.833λ (20)	10.5	12.5	13.5	n/a	59	n/a	n/a	n/a	n/a	n/a	0.366λ (50.76)	0.785λ${}^{2}$ × 0.366λ	FDM	n/a
[22]	5.77 to 6.24	12.56λ${}^{2}$ (Radius = 100 mm)	0.46λ (23)	5.77	6	17	n/a	7.8	22.2	n/a	n/a	Linear	n/a	0.04λ (2)	12.56λ${}^{2}$ × 0.04λ	Photo polymer resin with surface metalization	n/a
[23]	X−band (λ = 28.3 mm)	2.86λ${}^{2}$ (Radius = 27 mm)	0.96λ (27.2)	10	10.6	15	16.048	49.65	28.6	n/a	−10.4 in 10.6 GHz	Linear	699	0.477λ (13.5)	2.86λ${}^{2}$ × 0.477λ	FDM	16.4
Proposed	10 to 15	2λ (50) × 2λ (50)	1.17λ (29.33)	10	12	17.2	17.6	50	20	−15.1 in 10 GHz, −16.9 in 12.5 GHz, −16.7 in 15 GHz	−13.7 in 10 GHz, −14.2 in 12.5 GHz, −11.5 in 15 GHz	Linear	860	0.49λ (12.33)	2λ × 2λ × 0.49λ	FDM	79 g, copper painted, 96 g with PREPERM10
